# Effects of high intensity speed-based treadmill training on ambulatory function in people with chronic stroke: A preliminary study with long-term follow-up

**DOI:** 10.1038/s41598-018-37982-w

**Published:** 2019-02-13

**Authors:** Sangeetha Madhavan, Hyosok Lim, Anjali Sivaramakrishnan, Pooja Iyer

**Affiliations:** 1 Brain Plasticity Laboratory, Department of Physical Therapy, University of Illinois at Chicago, Chicago, IL USA; 2 Graduate Program in Rehabilitation Sciences, College of Applied Health Sciences, University of Illinois at Chicago, Chicago, IL USA

## Abstract

High intensity treadmill training has shown to be beneficial for stroke survivors, yet the feasibility and long-term effects remain unclear. In this study, we aimed to determine whether a 4-week high intensity speed-based treadmill training (HISTT) is feasible for chronic stroke survivors, and we examined its effects on ambulatory function, and long-term retention. Sixteen individuals post-stroke participated in 40 minutes of HISTT for four weeks at a frequency of three sessions per week. Gait speed was measured using the 10-meter walk test, endurance was measured using the 6-minute walk test, and quality of life was assessed using the Stroke Impact Scale (SIS) at baseline, post-training, and at 3-month follow-up. All participants successfully completed the training without any serious adverse events. Participants significantly increased fastest walking speed by 19%, self-selected walking speed by 18%, and walking endurance by 12% after the training. These improvements were maintained for 3 months after the intervention. Our results indicate that this modified speed-based high intensity walking program has the potential to be a feasible and effective method of gait training for stroke survivors. However, the small sample size and lack of a control group warrant caution in interpretation of results. Further studies are recommended to better understand effectiveness of this protocol in combination with other physical therapy interventions for functional recovery after stroke.

## Introduction

A large proportion of stroke survivors (up to 70%) experience considerable gait deficits, including reduced walking speeds and asymmetrical walking patterns, limiting their capacity for community ambulation^1,2^. An average walking speed of 1.32 m/s is required for safe and independent ambulation in the community^3^. However, the average walking speed of community-dwelling stroke survivors ranges within 0.3 to 0.8 m/s^4,5^. This highly reduced walking speed is a critical barrier for stroke survivors to fully assimilate in the community. Compromised walking endurance post-stroke is reported to be significantly associated with limited community participation^6^. Restricted community participation encourages a sedentary lifestyle leading to increased risk of cardiac events or recurrent stroke and reduced quality of life^7–9^. Thus, there is a critical need to enhance gait training outcomes post-stroke, especially walking speed and endurance.

Treadmill training has long been utilized as an effective and feasible method of gait training for post-stroke individuals^10^. The popularity of treadmill training post-stroke is based on its easy clinical and home accessibility, task-specificity, and high repetition which facilitates improved ambulatory and cardiovascular function^11–13^. High-intensity interval training (HIT) is an increasingly popular exercise training program that maximizes exercise intensity by the combination of short bursts of maximal effort alternated with longer recovery periods. Growing evidence suggests that HIT demonstrates superior health outcomes when compared to moderate intensity training in healthy adults, people with cardiovascular disorders, and stroke survivors^14–19^. Moreover, training at high intensity requires up to 40% less time commitment than training at moderate intensity^20^.

Speed-based HIT is one type of HIT that is primarily designed to improve an individual’s walking speed by training at the maximum tolerated treadmill belt speed^19,21–23^. Although speed-based HIT and conventional HIT have much in common, discrepancy exists in prescribing intensity. Speed-based HIT channels intensity based on one’s ability to walk at the maximum belt speed, whereas the conventional HIT uses heart rate (HR). A recent study reported that speed-based HIT yields greater aerobic capacity and fatigue resistance in female athletes when compared to HR-based HIT^24^. Only three studies have performed long term speed-based HIT in individuals with stroke. Significant improvements in overground walking speed, stride length, and aerobic capacity were noted after two to four weeks of training in subacute and chronic stroke survivors^19,21,22^. However, the effect of speed-based HIT on other gait parameters, quality of life, and long-term retention has not been investigated before.

In this study, we present a modified speed-based HIT adapted from the treadmill training protocol proposed by Pohl and colleagues^21^. We have previously reported the effects of a single session of this modified high intensity speed-based treadmill training (HISTT) on cortical excitability in stroke survivors^25^. The goals of the present study were to assess the safety and feasibility of a 4-week HISTT protocol in people with chronic stroke, with long-term follow up at 3-months. We also examined the effects of HISTT on ambulatory function.

## Results

### Safety and Feasibility

**Figure 1:**
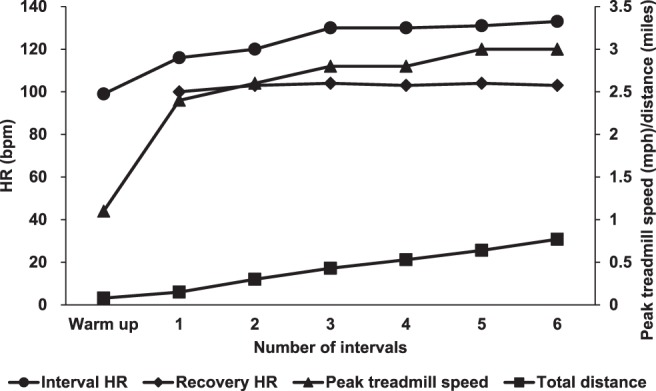
Representative example from one participant during a single session of high intensity speed-based treadmill training (HISTT). Interval heart rate (*circles*), recovery heart rate (*diamonds*), highest treadmill speed achieved during each interval (*triangles*), and accumulated distance walked (*squares*) are shown. Heart rate (beats per minute) is represented on the left primary y-axis. Peak treadmill speed (miles per hour) and distance (miles) are represented on the right secondary y-axis. The x-axis represents the number of intervals achieved.

A total of 16 participants with stroke were initially admitted to the study. Participants were questioned for serious and non-serious adverse events at the beginning and end of every session. A serious adverse event was defined as an event that results in severe health conditions which may necessitate medical or surgical treatment. A non-serious adverse event was defined as an event that results in minor discomfort (muscle soreness, fatigue etc.) which does not require session/study termination. All participants were able to tolerate the 4-week HISTT without any orthopedic, cardiovascular, or other serious adverse events. About five out of 16 participants indicated that the training resulted in muscle soreness and fatigue after the end of the session, but they were able to continue training the next session. No drop-outs were observed during training. On average, participants started at a treadmill belt speed of 0.84 ± 0.30 m/s and were capable of increasing the belt speed from three to five 10% increments. A representative example of HR, peak treadmill speed, and distance achieved from one participant during a single training session is presented in Fig. 1.

### Clinical outcome measures

**Figure 2:**
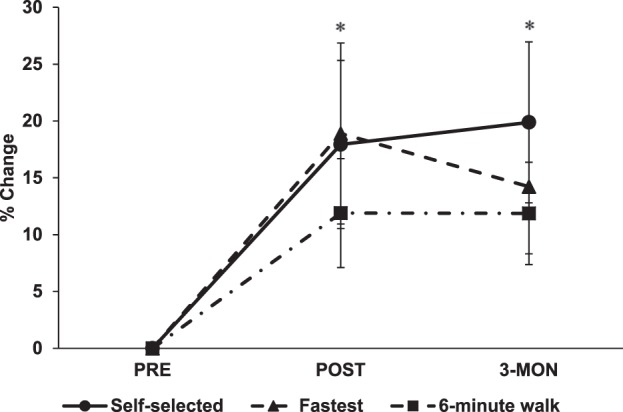
Average percent changes of self-selected walking speed (*circles*), fastest walking speed (*triangles*), and 6-minute walking distance (*squares*) from PRE to POST and 3-MON. Error bars indicate standard error of mean. *Significantly different than PRE (p < 0.05).

A one-way repeated measures ANOVA revealed statistical significance for fastest walking speed (F_2,30_ = 6.97, p = 0.003), self-selected walking speed (F_2,30_ = 7.13, p = 0.003), walking endurance (F_2,30_ = 3.86, p = 0.032), and overall SIS (F_2,30_ = 4.11, p = 0.026) (Table 1). Post hoc analysis revealed significant differences from PRE to POST, and from PRE to 3-MON for all variables. The fastest walking speed showed a significant improvement of 19% from PRE to POST (p = 0.006), and 14% from PRE to 3-MON (p = 0.021). The self-selected walking speed significantly increased by 18% from PRE to POST (p = 0.013) and 20% from PRE to 3-MON (p = 0.009). Walking endurance showed 12% improvement from PRE to POST (p = 0.054) and 12% from PRE to 3-MON (p = 0.030). Percent changes for the walking tests are presented in Fig. 2. The SIS revealed 6% improvement from PRE to POST (p = 0.001) and 8% from PRE to 3-MON (p = 0.046).

**Table 1:** Changes in walking speed, endurance, and health-related quality of life before and after HISTT.

	PRE	POST	3-MON
10 m walk test (m/s)			
Self-selected speed	0.80 ± 0.20	0.91 ± 0.19*	0.94 ± 0.21*
Fastest speed	1.04 ± 0.30	1.18 ± 0.28*	1.16 ± 0.31*
6-minute walk test (m)	299.08 ± 76.97	326.43 ± 67.18*	327.44 ± 65.96*
Stroke impact scale	546.85 ± 21.99	579.73 ± 21.05*	588.17 ± 22.02*

Data are presented as mean ± SD. HISTT, high intensity speed-based treadmill training; m, metres; s, seconds. *Significantly different than PRE (p < 0.05).

### Treadmill and cardiovascular measures during training

**Figure 3:**
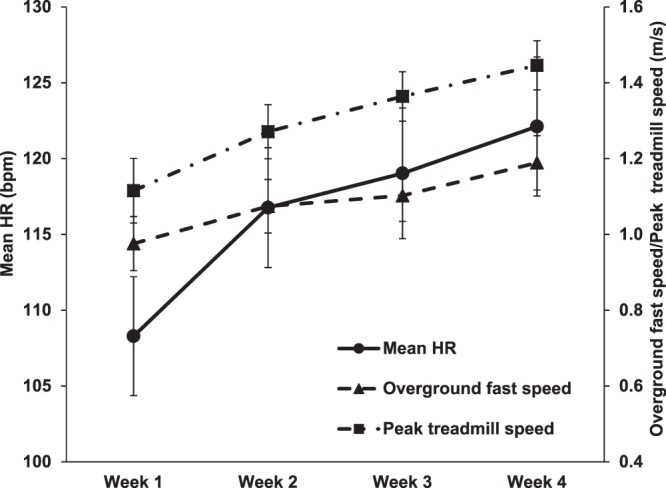
Changes in mean heart rate (*circles*), overground fastest walking speed (*triangles*), and peak treadmill speed (*squares*) by week. Mean heart rate (beats per minute) during speed-based intervals is represented on the left primary y-axis. Overground fastest speed and peak treadmill speed (metres per second) are represented on the right secondary y-axis. The x-axis represents the four different weeks. Error bars indicate standard error of mean.

A one-way repeated measures ANOVA revealed statistical significance for the weekly measurements such as overground fastest walking speed (F_3,45_ = 12.52, p < 0.001), peak treadmill speed (F_1.93,28.95_ = 69.02, p < 0.001) and mean HR (F_3,45_ = 27.83, p < 0.001). Post hoc analysis revealed that the participants significantly increased overground fastest walking speed compared to week 1: 13% at week 3 (p = 0.033) and 22% at week 4 (p = 0.001). Significant improvement was found at week 4 compared to weeks 2 and 3 by 11% and 8%, respectively (p = 0.028; p = 0.007). Peak treadmill speed significantly increased by 14% at week 2, 22% at week 3, and 30% at week 4 when compared to week 1 (all p < 0.001). Peak treadmill speed was also significantly improved by 7% at week 3 and 14% at week 4 when compared to week 2 (all p < 0.001). Significant improvement by 6% at week 4 was found when compared to week 3 (p = 0.002). Mean HR and peak HR, substantially increased by 8% at week 2, 10% at week 3, and 12% at week 4 when compared to week 1 (all p < 0.001). However, no significant difference was found between week 2, week 3, and week 4 (Table 2). Weekly changes of overground fastest walking speed, peak treadmill speed, and mean HR are presented in Fig. 3.

**Table 2:** Changes in overground fastest walking speed, peak treadmill speed, and heart rate measurements by week.

	Week 1	Week 2	Week 3	Week 4
OG fastest speed (m/s)	0.98 ± 0.28^c,d^	1.07 ± 0.28^d^	1.10 ± 0.27^a,d^	1.19 ± 0.28^a,b,c^
Peak TM speed (m/s)	1.12 ± 0.34^b,c,d^	1.27 ± 0.28^a,c,d^	1.36 ± 0.26^a,b,d^	1.45 ± 0.25^a,b,c^
Peak HR (bpm)	114.50 ± 17.77^b,c,d^	123.31 ± 16.64^a^	125.42 ± 19.08^a^	128.67 ± 19.52^a^
Mean HR (bpm)	108.29 ± 15.69^b,c,d^	116.77 ± 15.8^a^	119.03 ± 17.22^a^	122.12 ± 18.35^a^
Percentage of age-predicted maximum HR (%)	66.96 ± 10.40	72.11 ± 9.74	73.34 ± 11.16	75.24 ± 11.42

OG, overground; TM, treadmill; HR, heart rate; m, metres; s, seconds; bpm, beats per minute. Data are presented as mean ± SD. ^a^Significantly different than week 1 (p < 0.05). ^b^Significantly different than week 2 (p < 0.05). ^c^Significantly different than week 3 (p < 0.05). ^d^Significantly different than week 4 (p < 0.05).

## Discussion

This study aimed to evaluate the safety and feasibility of 4-week HISTT protocol in individuals post-stroke. All participants completed the 12 sessions of training with no serious adverse events reported during the training period. After 4-weeks of HISTT, stroke survivors demonstrated significant speed and endurance related improvements in walking, reported improvements in quality of life, and retained these improvements up to 3-months after training.

A total of 192 treadmill training sessions were successfully completed without any reports of serious adverse events or patient dropouts. None of the participants engaged the safety harness during training (i.e. no stumbles or trips were experienced). Excessive changes in HR, defined as more than 10% changes in HR above the upper cut-off safety limit, and abnormal changes in BP were not observed during the training period. Fifteen participants tolerated the training for 37–40 minutes during each session. One participant required multiple recovery pauses during each session due to fatigue, which resulted in training duration of 28–36 minutes per session. Eleven out of 16 participants reached close to 80% of age-predicted maximum HR for all 12 sessions while three others reached this level during the third week of training. One participant who was on beta blockers maintained a steady HR despite the significant increase in peak treadmill speed. One participant’s HR was maintained between 53–70% age-predicted maximum HR during the entire training period. Average RPE score during the intervals ranged from 5.3 to 6.3 (out of 10) which represents ‘heavy’ subjective rating of exertion. Although there was initial hesitation and apprehension for walking at high speeds, patients reported enjoying the training and many suggested they would like to continue further if possible. This is also evident with significant increase seen in the overall SIS, which reflects an improvement in perceived health status after the intervention.

Twelve sessions of HISTT resulted in significant improvements in walking outcomes which remained greater than baseline at 3-months after training. Immediately after training, a 0.14 m/s improvement in fastest walking speed and a 0.11 m/s in self-selected walking speed was noted, which are clinically relevant according to the minimal clinically important differences (MCID) scale established for walking speed which is from 0.10 to 0.20 m/s and can be considered a meaningful change^26,27^. The changes in walking endurance (27.36 m) was also clinically significant according to the established MCID range for the 6-minute walk test which is from 14.0 to 30.5 m^26,28^.

Only three previous studies have utilized a speed-based treadmill training protocol to optimize overground walking speed in people post-stroke^19,21,22^. Pohl *et al*. (2002) found a gain of 1.02 m/s (0.61 to 1.63 m/s) in fastest walking speed after a 4-week speed-based training protocol^21^. Lau and Mak (2011) reported improvements in fastest walking speed by 0.36 m/s (0.28 to 0.64 m/s) after four weeks of speed-based training^22^. Boyne *et al*. (2016) reported improved fastest walking speed by 0.1 m/s (0.77 to 0.87 m/s) after 4-week speed-based treadmill training^19^. In the Pohl *et al*. (2002) and Lou and Mak (2011) studies, participants received an additional 45–90 minutes of conventional gait rehabilitation after the speed-based treadmill training. This additional dosage in treatment may have contributed to greater improvements in these two studies compared to ours. In addition, these studies also recruited stroke survivors in relatively early stages of stroke (4.05 & 0.43 months post-stroke respectively) compared to the current study where we intentionally recruited chronic stroke survivors (76.44 months post-stroke) to establish safety and feasibility. Considering that up to 91% of motor recovery occurs within three months after the onset of stroke, higher motor achievements in the two previous studies may perhaps be explained^29^. We also noticed that Pohl *et al*. (2002) and Lau and Mak (2011) reported lower baseline fastest walking speed of 0.61 and 0.28 m/s respectively compared to the higher 1.04 m/s gait speed in our study, possibly resulting in a ceiling effect^21,22^. We report a slightly higher improvement in fastest walking speed (0.14 m/s) compared to Boyne *et al*.^19^. The protocol applied by Boyne *et al*.^19^ involved walking at the highest tolerated speed for 30 seconds with 0.1 mph increment after completing the interval successfully or 0.1mph decrement after exhibiting mechanical fault during the interval. Their study also incorporated stationary recovery periods while we used an active recovery paradigm. Differences in the training protocol and sample cohort may explain the difference in gait speed improvements between this study and ours.

Participants in our study maintained or further improved the achieved gain in ambulatory function even three months after HISTT. This finding is important since, to our knowledge, there are no previous studies that have reported the retention effect of speed-based HIT. A possible explanation for the retention effect could be attributed to the increased community participation attained with improvements in walking speed. Significant improvement in perceived health-related quality of life may have also encouraged the participants to actively engage in community activities and thus retain improvement^30^.

The speed-based HIT studies appear to yield greater speed improvements when compared to the conventional HIT studies^13,31^. Globas *et al*. (2012) reported significant improvement in fastest walking speed (0.12 m/s) and self-selected walking speed (0.07 m/s) after 3-months of HIT in stroke survivors^13^. Greater improvements shown in the current study can be considered substantial due to the relatively short duration of training (4 weeks). Similar improvements for fastest walking speed (0.14 m/s) was reported by Gjellesvik *et al*. (2012), however, greater number of sessions were conducted when compared to our study (20 vs 12 sessions)^31^. In addition, the average highest achieved belt speed of 1.50 m/s in the present study was greater than 1.01 m/s reported from the conventional HIT study^13^. It should be noted that gain achieved on the treadmill did not directly translate to the overground environment. Differences in mode of walking and gait biomechanics between the treadmill and overground walking are potential factors that may affect transference^32^.

The improvement in ambulatory function obtained with HISTT may be explained not only by the increased cardiovascular activity elicited with rapid stepping, but also to the potentially increased neuromuscular demand to maintain progressive fast walking. Increased paretic-leg propulsive force and reduced interlimb asymmetry are found with high speed treadmill walking in individuals with stroke^33^. Since our participants were continuously trained to walk at the maximum belt speed every session, the changes in gait biomechanics and repetitive massed practice may have contributed to significant improvements in ambulatory function. Future studies are recommended to assess the effect of speed-based treadmill intervention on gait biomechanics in stroke survivors.

We recognize some important limitations in this study that must be taken into consideration. First, a small sample size limits the generalizability and interpretation of our findings. Moreover, our randomly selected sample included stroke survivors with relatively high functional mobility. Thus, this protocol may need to be modified or may not be applicable to household ambulators or individuals with limited community ambulation. Second, we did not include a control group. Although our findings demonstrated clinically relevant improvements in overground walking speed and endurance, we do not know if this modified protocol is superior to other treadmill training protocols that have been previously reported. Thirdly, we did not include an exercise stress test at the start of the training or continuously monitor electrocardiography or BP during the training. This may be necessary to warrant safety of participants who are cardiovascularly compromised. Lastly, we included a two-minute active recovery period between the speed-based intervals. The length of interval time may have resulted in suboptimal training or recovery dosage for some participants^23^.

The present study supports the safety and feasibility of 4-weeks of HISTT and provides preliminary evidence that HISTT may have the potential to improve gait speed, endurance, and quality of life of chronic stroke survivors, and these improvements are maintained likely up to 3-months after training. This study is one of the few speed-based training protocols that demonstrates the safety, feasibility, and ambulatory changes in chronic stroke, without the need for expensive equipment such as body weight support systems. Future larger studies are needed to understand the potential mechanisms of HISTT on gait biomechanics and cardiovascular function post stroke.

## Methods

### Study design

A single group pre- and post-test intervention design with a 3-month follow-up was conducted in chronic stroke participants as a part of a larger ongoing randomized controlled trial (RCT) at the Brain Plasticity Laboratory (clinical trial registration: NCT03492229, 04/10/2018). All participants performed four weeks of HISTT (3 times/week, 40 minutes/session). Participants were required to attend three additional visits to perform clinical tests: pre-training (PRE), post-training (POST), and 3-month follow-up (3-MON).

### Participants

A total of 16 individuals post-stroke (10 males/6 females, mean age 57.4 ± 9.7 years) were included in this secondary analysis. This included all participants who completed the HISTT protocol by March 2018. Participant demographic information is presented in Table 3. Inclusion criteria for the ongoing RCT were as follows: (i) age 40–80 years; (ii) first ever mono-hemispheric stroke; (iii) minimum of six months post-stroke; and (iv) ability to perform independent walking for at least five minutes with or without an assistive device. Participants were excluded from the study if they had any of the following: (i) chronic cardiorespiratory or metabolic diseases; (ii) significant cognitive or communication impairment (Mini Mental State Examination score < 21); (iii) severe osteoporosis; and (iv) lesions pertaining to the brainstem or cerebellum. The study was approved by the Institutional Review Board of the University of Illinois at Chicago and conformed to the Declaration of Helsinki. Written informed consent was obtained from all participants prior to the beginning of the study.

**Table 3:** Participant characteristics.

Participant Charecteristics	
Gender (Male/Female)	10/6
Age (years)	57.44 ± 9.77
Type of stroke (Ischemic/Hemorrhagic)	13/3
Time since stroke (years)	6.37 ± 4.53
Side of hemiparesis (Right/Left)	5/11
FMA-LE	21.19 ± 5.26

Data are presented as mean ± SD. FMA-LE, Fugl-Meyer assessment - lower extremity.

### Clinical outcome measures

Walking speed was evaluated with the 10-meter walk test (10MWT)^34,35^. The test was performed at the fastest and self-selected walking speeds for the marked distance. Fastest and self-selected walking tests were conducted twice, and the average was used in the analyses. Walking endurance was assessed by the 6-minute walk test (6MWT)^36^. Participants were instructed to walk as far as they can for six minutes. The Stroke Impact Scale (SIS) was used to measure health-related quality of life^37^. All clinical measurements were performed at PRE, POST and 3-MON time points. In addition, overground fastest walking speed was recorded at the beginning of every week (Week 1, Week 2, Week 3 and Week 4).

### Treadmill and cardiovascular measures during training

Peak treadmill speed was defined as the highest belt speed at which the participant was able to walk safely and without stumbling. HR was recorded continuously during the training using a HR monitor (Polar H7, Polar Electro Inc., Kempele, Finland). The HR achieved at the end of each interval or recovery period was considered as the interval HR or recovery HR respectively and a mean was calculated. The highest HR reached within a session was considered as the peak HR. Rating of Perceived Exertion (RPE) was monitored after each interval using the 10-point modified Borg Scale^38^.

### HISTT protocol

The primary goal of the HISTT was to continuously achieve increased maximum belt speeds in every session. A single session involved 40 minutes of treadmill walking: 5-minute warm-up, 30-minute high intensity speed-based intervals interleaved with active recovery, and a 5-minute cooldown. The treadmill was fixed at 0% incline and no manual assistance was given, although one research assistant was positioned behind the participant in case of a trip or fall. Participants were fastened in an overhead harness for safety with no body weight support. Participants were allowed to hold onto the handrails for support, however, minimal use of handrail was encouraged. HR and RPE were monitored throughout the training session for any signs of cardiovascular intolerance. Blood pressure (BP) was measured before and after training, and as needed if the participants showed signs of pallor, excessive sweating or dizziness. Age-predicted maximum HR determined using the formula (220 − age) and HR achieved during warm-up were used as reference values while increasing the belt speed. Safety criteria for stopping the session included: (i) excessive increase in HR above 80% of age-predicted maximum HR, (ii) failure to recover HR to warm-up level during recovery, (iii) hypertensive response (systolic BP > 200 mm Hg or diastolic BP > 110 mm Hg), (iv) verbal or physical manifestations of severe fatigue, light headedness, (v) development of paleness and excessive sweating or confusion, and (vi) onset of angina or inappropriate bradycardia^39^.

**Figure 4:**
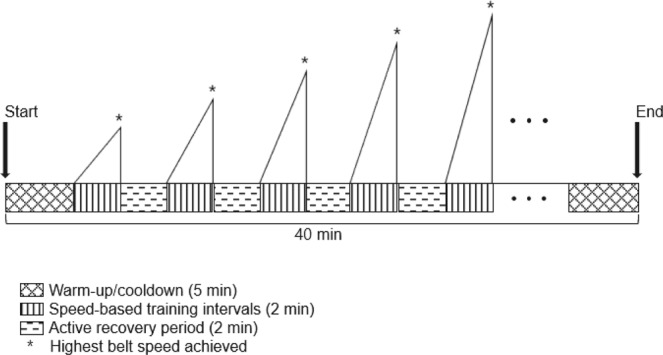
Schematic of a single session high intensity speed-based treadmill training (HISTT). HISTT involved 40 minutes of treadmill walking: 5-minute warm-up, 30-minute speed-based training intervals, and 5-minute cool down. The training consists of multiple 2-minute speed-based intervals alternated with 2-minute active recovery periods. During the speed-based training intervals, the belt speed is gradually increased to the participant’s tolerable highest speed (*) and maintained for 10 seconds. The recovery requires the participant to walk at warm-up/cool down speeds until the heart rate recovers to warm-up level.

For the first training visit, overground fastest walking speed was measured using the 10MWT. The obtained speed was then halved and used for the 5-minute warm-up phase on the treadmill. After the warm-up, the first speed-based training interval was initiated. During a period of two minutes, the belt speed was slowly increased, within the participant’s tolerance, to the highest speed at which the participant could walk safely and without stumbling. At the end of the two-minute interval, this maximum achieved belt speed was held for ten seconds. This was followed by a recovery period when the participant walked at the warm-up speed until a time at which the participant’s HR and RPE returned to the levels reached during the warm-up phase. The next interval began if the HR recovered to the level achieved during the warm-up. If the HR level was not restored, additional active recovery time or rest was given until the HR reduced to the warm-up level. If the participant maintained the speed and felt safe during the ten seconds at the end of the first-training interval, the speed was then increased by 10% during the next interval. During any fast walking phase, if the participant was unable to maintain the required speed and felt unsafe, or if the HR reached the cut-off safety limit, the speed was then reduced by 10% for the next interval. At the end of thirty minutes of structured walking, a 5-minute cooldown phase was provided. A design of the HISTT protocol is shown in Fig. 4.

### Statistical analysis

A one-way repeated measures ANOVA was used to compare changes between PRE, POST and 3-MON for the fastest walking speed, self-selected walking speed, walking endurance, and overall SIS. A one-way repeated measures ANOVA was used to compare the weekly differences (Week 1, Week 2, Week 3 and Week 4) for the overground fastest walking speed, peak treadmill speed achieved during the interval every week, mean HR and peak HR. Significant main effects and interactions were followed up with post-hoc tests. Statistical significance was set at *p* < 0.05. Values are reported as mean ± standard deviation. All statistical analyses were conducted using SPSS software, version 24.0 (IBM SPSS Statistics 24, Armonk, NY, USA).
